# Advanced Nanoparticle-Based Drug Delivery Systems and Their Cellular Evaluation for Non-Small Cell Lung Cancer Treatment

**DOI:** 10.3390/cancers13143539

**Published:** 2021-07-15

**Authors:** Noratiqah Mohtar, Thaigarajan Parumasivam, Amirah Mohd Gazzali, Chu Shan Tan, Mei Lan Tan, Rozana Othman, Siti Sarah Fazalul Rahiman, Habibah A. Wahab

**Affiliations:** 1 School of Pharmaceutical Sciences, Universiti Sains Malaysia, Gelugor 11800, Penang, Malaysia; noratiqah@usm.my (N.M.); thaigarp@usm.my (T.P.); amirahmg@usm.my (A.M.G.); weazley90@hotmail.com (C.S.T.); tanml@usm.my (M.L.T.); habibahw@usm.my (H.A.W.); 2 Faculty of Pharmacy, Universiti Malaya, Kuala Lumpur 50603, Malaysia; 3 Center for Natural Products Research and Drug Discovery (CENAR), Universiti Malaya, Kuala Lumpur 50603, Malaysia

**Keywords:** non-small cell lung cancer, drug delivery, nanoparticles, pulmonary, cellular uptake, permeability, A549 cell line, cytotoxicity, MTT assay

## Abstract

**Simple Summary:**

Nanoparticulate systems have been extensively explored for the treatment of various diseases, including cancers. The outstanding characteristics of nanoparticles have made it possible to administer them via different routes such as intravenous or inhalation. This flexibility can improve the delivery of encapsulated drugs to the targeted cells for the treatment of lung-related diseases and cancers such as non-small cell lung cancers. The effectiveness of a treatment option needs to be validated in suitable in vitro and/or in vivo models. As the handling of in vivo models is a challenge, many researchers have turned towards in vitro models that use normal cells or specific cells from diseased tissues. This review focuses on the currently available nanoparticles for lung cancers and the type of cellular work that can be conducted to evaluate the effectiveness of a nanoparticulate system for this cancer type.

**Abstract:**

Lung cancers, the number one cancer killer, can be broadly divided into small cell lung cancer (SCLC) and non-small cell lung cancer (NSCLC), with NSCLC being the most commonly diagnosed type. Anticancer agents for NSCLC suffer from various limitations that can be partly overcome by the application of nanomedicines. Nanoparticles is a branch within nanomedicine that can improve the delivery of anticancer drugs, whilst ensuring the stability and sufficient bioavailability following administration. There are many publications available in the literature exploring different types of nanoparticles from different materials. The effectiveness of a treatment option needs to be validated in suitable in vitro and/or in vivo models. This includes the developed nanoparticles, to prove their safety and efficacy. Many researchers have turned towards in vitro models that use normal cells or specific cells from diseased tissues. However, in cellular works, the physiological dynamics that is available in the body could not be mimicked entirely, and hence, there is still possible development of false positive or false negative results from the in vitro models. This article provides an overview of NSCLC, the different nanoparticles available to date, and in vitro evaluation of the nanoparticles. Different types of cells suitable for in vitro study and the important precautions to limit the development of false results are also extensively discussed.

## 1. Introduction

After breast cancer, lung cancer (LC) is the second most diagnosed cancer in the world, with over two million new cases in 2020 [1]. However, with 1,796,144 deaths or 18% of 9,958,133 total cancer deaths, lung cancer appears as the top cancer killer in the world [1]. The chief cause of lung cancer is cigarette smoking, but there are significant geographical and racial influences on lung cancer cases [2,3]. In general, lung cancer can be categorized into small cell lung cancer (SCLC) and non-small cell lung cancer (NSCLC). SCLC is a lethal tumor accounting for approximately 15% of lung cancers, while NSCLC accounts for about 80–85% of lung cancers.

NSCLC can be further categorized into three types, namely, adenocarcinomas, squamous cell carcinomas and large cell carcinomas. Lung adenocarcinoma occurs in the lung periphery, usually related to the surface alveolar epithelium or bronchial mucosal glands, accounting for about half of all lung cancers [4]. Although it develops more often in smokers, this type of lung cancer is also common among non-smokers [5]. Squamous cell lung cancer is usually located in the lung’s center and arises in the proximal bronchi. It also develops in smokers and typically represents around 25% to 30% of NSCLC [6]. In contrast, large cell carcinoma can appear anywhere in the lung tissue and grows more rapidly than adenomas or squamous cell lung carcinomas. Although it accounts for approximately 3% of all lung cancers, the overall survival in patients with large cell carcinoma is significantly worse than the other subtypes [7].

Current treatment options for NSCLC include surgery, targeted therapy, chemotherapy, and radiation therapy [3]. Systemic treatments (chemotherapy, targeted therapy, or immunotherapy) are required for the vast majority of patients (clinical stages Ib–IV) [8]. However, the therapy selection, dosing, and administration are evolving and complex [9] and beyond the scope of this review. In general, the first-line treatment for NSCLC typically is platinum-based chemotherapy: cisplatin or carboplatin doublets [10,11]. Platinum-based chemotherapy has a low therapeutic ratio, with significant toxicities including severe nausea and vomiting, renal toxicity requiring adequate hydration, ototoxicity, and neuropathy. Cisplatin-containing regimens have also been shown to be more toxic than those without it [11,12]. Since NSCLC is typified by several gene point mutations and about 70% of NSCLC patients experience somatic mutations in the exons of the epidermal growth factor receptor (EGFR) gene [13,14], small-molecule EGFR tyrosine kinase inhibitors (EGFR-TKIs) such as erlotinib and gefitinib have been included as the second-line treatment for NSCLC. As compared with the standard chemotherapeutic regimen, EGFR-TKIs significantly improve objective response rate, progression-free survival, and quality of life and show mild toxicity. In fact, the newest EGFR-TKI, osimertinib, since its approval in April 2018, has been widely adopted as first-line therapy for patients with advanced EGFR-mutant NSCLC [15]. This remarkable advance in the use of EGFR-TKIs in the treatment of NSCLC is currently gaining impact in the area of targeted therapy and precision medicine.

Other treatment options for patients with metastatic NSCLC who progress after platinum-based chemotherapy (second line and beyond) include pemetrexed and docetaxel [16] as well as anti-program cell death protein-1 (PD-1) inhibitors such as nivolumab, pembrolizumab and programmed cell death ligand-1, atezolizumab [10]. Pembrolizumab, in 2017, was approved by US FDA as the first-line treatment for patients with metastatic NSCLC with high PD-1 expression [10]. Very recently, the FDA also approved the combination of nivolumab and ipilimumab (the cytotoxic T-lymphocyte-associated antigen-4 (CTLA-4) inhibitor) as the first-line treatment for certain patients with metastatic or recurrent NSCLC, with no EGFR or anaplastic lymphoma kinase (ALK) genomic tumor aberrations [17]. These immune checkpoint inhibitors (ICI) presented a new landmark in oncology, and they have been shown to be significantly better than the use of docetaxel in terms of overall survival, progression-free survival, duration of response, and overall response rate [18]. Although all these drugs are highly effective in treating NSCLC, they still suffer from several limitations, including toxicity, severe side effects, drug resistance problems, high dose requirements for efficacy, and high treatment costs. Several approaches have been explored to overcome these limitations. One promising approach includes the utilization of nanotechnology for cancer treatment, which has been shown to substantially reduce the cost, increase the therapeutic efficacy, and reduce systemic toxicity [19].

Nanomedicine is a nanotechnology branch that employs materials between 5–200 nm, applied to health and medicine [20]. Within nanomedicine, a nanoparticle drug delivery system (NDDS) is a potential solution for overcoming the limitations of anti-cancer drugs, as it can improve the effectiveness of the drugs by increasing the stability and bioavailability, improving transport across the biological barriers and disease targeting, prolonging circulation and blood concentration, reducing enzyme degradation, and reducing the toxicity and immunogenicity of the drugs [21,22]. Nanoparticles are also advantageous to augment the accumulation of therapeutic agents in the cancer tissues via an enhanced permeability and retention (EPR) effect [23,24,25]. Many types of nanoparticles have been formulated for the delivery of anti-cancer agents, although only a handful has reached the clinical stage.

Among the challenges for progress in NDDS is the requirement for thorough physicochemical characterization and evidence of safety and efficacy in the delivery of the encapsulated drug to cancer cells [26]. Predictive in vitro models of cancer cells or tissues are required to assess efficacy, absorption, and uptake as well as safety. It is unlikely that any in vitro model would be sufficient as a final decision point for safety and efficacy, but a useful in vitro toxicity screening model should be well-characterized and predictive of in vivo effects with a low incidence of false positives or false negatives [27].

Based on the data available in the literature, NDDS has a high potential for lung delivery of active pharmaceutical ingredients (API). Due to their nanosize, these nanocarriers may be capable of effectively traversing the bronchial epithelium barrier and accumulating in deep lung regions. Herein, we present an overview of the latest nanotechnological approaches for the delivery of chemo-immunotherapeutic agents against NSCLC. Types of NDDS, use of cell evaluation, and the rationale behind their applications in permeability and cell uptake studies as well as toxicity evaluation of the NDDS are also presented and discussed.

## 2. NDDS for Chemo- and Immunotherapy against NSCLC

Various nanocarrier platforms are being investigated as the drug delivery systems for an arsenal treatment of NSCLC. Lipid-based (i.e., liposomes, solid lipid nanoparticles (SLNs) and nanostructured lipid carriers (NLCs)), polymeric-based, and metal-based nanoparticles are some of the well-studied classes of nanocarriers. They are briefly discussed in the following subsections.

### 2.1. Liposomes

**Figure 1:**
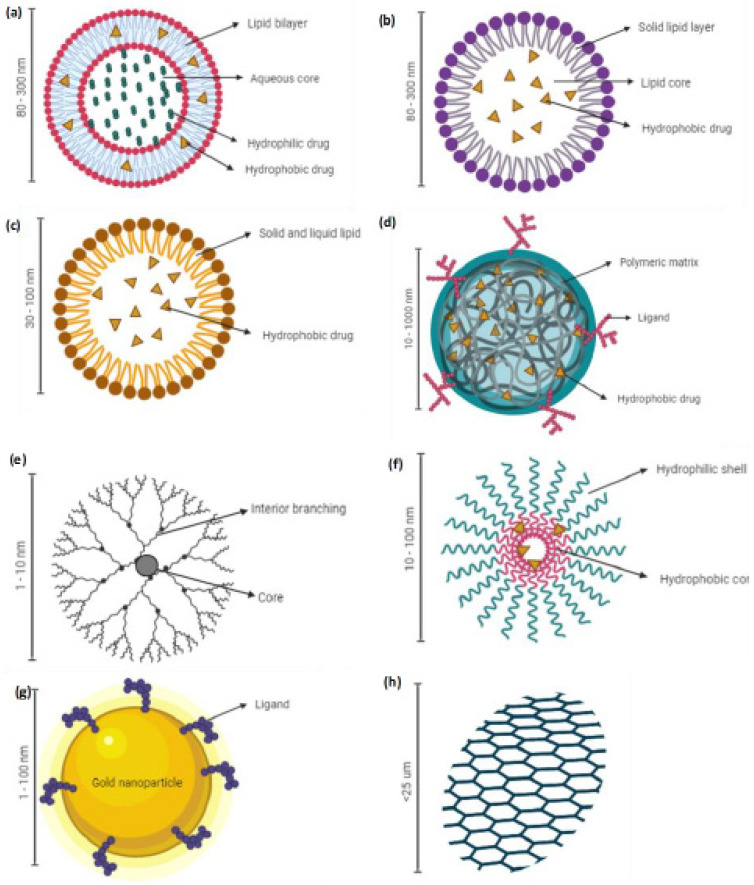
Schematic representation of various nanocarriers experimented for the treatment of non-small cell lung cancer with their respective particle sizes. (**a**) Nanoliposomes, (**b**) solid lipid nanoparticles, (**c**) nanostructured lipid carriers, (**d**) polymeric nanoparticles, (**e**) dendrimers, (**f**) polymeric micelles, (**g**) gold nanoparticles, and (**h**) graphene (created using BioRender.com, accessed on 2 April 2021).

Doxil is the first nanoliposome formulation containing doxorubicin approved by the US FDA in 1995 to treat AIDS-related Kaposis sarcoma [28]. Liposomes are spherical delivery vehicles consisting of amphiphilic lipid bilayers that can entrap hydrophilic molecules in the aqueous core and hydrophobic molecules within the lipid bilayers, as shown in Figure 1a. They resemble the phospholipid bilayer membranes, biocompatible, weakly immunogenic, and biodegradable, making them an efficient carrier system for drug delivery [29,30,31]. Nanoliposomes could also enhance the stability of the incorporated drugs in vivo, prolong the drug circulation, and readily modify the size and surface to improve the drug’s therapeutic index [32]. In addition, the EPR effect of radioisotope-loaded liposomes has been clinically proven in metastatic breast cancer patients using positron emission tomography (PET) imaging [23,33]. The advantageous pharmacological properties of liposomal formulations and their clinical applications have been extensively reviewed by Beltran-Gracia et al. [34]. However, nanoliposomes have a few limitations, including expensive production costs, poor stability at room temperature due to leakage of the encapsulated drug during storage, and batch-to-batch variation [35]. Liposomes have also been reported to induce the upregulation of pro-inflammatory cytokines and the increase in liver enzymes upon intravenous administration of positively charged lipid nanoparticles in healthy C57BL/6 mice [36].

### 2.2. Solid Lipid Nanoparticles (SLNs)

Solid lipid nanoparticles (SLNs) are colloidal nanocarriers developed in the 1990s to overcome the limitations of emulsions, liposomes, and polymeric systems [37]. SLNs are made of solid lipid where the drug is either present on the surface or entrapped inside the solid core (Figure 1b) [38]. They are biocompatible, can withstand minor pressure (i.e., nebulization), and are less prone to enzyme degradation than other colloidal systems [39,40]. The system also negates the use of organic solvents, which ease the scale-up for large production and confer better protection to the entrapped chemotherapeutic agent [41,42,43]. Unfortunately, similarly to other colloidal systems, the SLNs suffer the drawback of poor drug loading in which the drug loading is limited by the solubility of the drug in the melted lipid phase and expulsion of the drug during the storage [38]. An extensive review of SLNs for drug delivery systems can be found in other publications [44,45].

### 2.3. Nanostructured Lipid Carriers (NLCs)

Nanostructured lipid carriers (NLCs) are second-generation lipid-based nanoparticles reported in the mid-1990s to mitigate the drawback associated with SLNs, as mentioned in Section 2.2 [46]. NLCs are made of solid and liquid lipids in which partially crystallized nano-sized lipid particles are dispersed in an aqueous phase containing an emulsifier (Figure 1c) [40]. This loosely packed crystalline system allows the entrapment of drug molecules, reduces the leakage of a drug during storage, and allows controlled release of the drug [47,48,49]. NLCs have also been reported to have favorable distribution in the organs, including lungs, that could enhance lung cancer as well as other cancer treatment regimens [24]. Low drug loading capacity due to the crystalline nature of the lipids and gelation in the dispersed phase due to the solid lipids’ polymorphism are the major drawbacks of the NLC system [49,50,51].

### 2.4. Polymeric Nanoparticles

Polymeric nanoparticles have been extensively researched for NSCLC therapy, with polymers such as poly-lactic-acid-co-glycolic acid (PLGA), poly-lactic-acid (PLA), chitosan, and polycaprolactone [52,53]. Polymeric nanoparticles (Figure 1d) offer numerous advantages for drug delivery including their properties of being able to be manipulated for controlled and sustained release, easy surface modification, easy nanosizing, readily cellular uptake, being able to bypass reticuloendothelial clearance, and the ability to encapsulate various active molecules (i.e., drugs, peptides, and oligonucleotides), besides being biocompatible and biodegradable [54,55]. They also have superior storage stability as compared to lipid-based formulations [56]. Though these systems are found to be promising for integrin-targeted therapy, the large-scale production could be cumbersome due to the complex processing methodology. Nonetheless, the PLGA nanoparticulate system has been shown to be non-toxic in various in vitro and in vivo studies, which makes it a promising polymer to be explored for the treatment of NSCLC [38,57].

### 2.5. Dendrimers

Dendrimers are a unique class of polymeric nanoparticles first reported in the late 1970s [58]. They are synthetic molecules with repeatedly branched and radially symmetrical three-dimensional structures, as shown in schematic Figure 1e [59]. They consist of a core with highly repeating units, which are covalently linked to a nucleus, and terminal chemical structures, which form the surface of the dendrimers [59]. Dendrimers are versatile polymers owing to their predictable molecular weight, nanosize, monodisperse nature, and suitability for the encapsulation of hydrophobic and hydrophilic chemotherapeutic agents [60]. Their multifunctional surface also eases surface modification for targeted delivery. Other than structural defects due to the terminal functional group that cannot always be reacted stoichiometrically, dendrimer usefulness is hampered by batch-to-batch variation and expensive production costs [59]. An extensive review of dendrimers as a drug delivery tool is reported elsewhere [61,62].

### 2.6. Polymeric Micelles (PMs)

Polymeric micelles (PMs) (10–100 nm) are produced by the self-assembly of copolymers or amphiphilic surfactants in water above its critical micellar concentration (Figure 1f). PMs consist of a hydrophobic inner core surrounded by a hydrophilic shell structure [63]. Hence, hydrophobic and amphiphilic drugs can be encapsulated in the core to control their release [64,65]. The hydrophilic shell stabilizes the core and allows the particle to bypass the reticular endothelial system [64,65]. This, in return, prolongs the particle circulation in the blood, which could enhance the particle accumulation in the tumor tissues. The major drawback of PMs is leakage of the encapsulated drug in the blood circulation and during storage [63,66]. A topical dosage form instead of intravenous administration could mitigate some of these limitations related to PMs.

### 2.7. Metal-Based Nanoparticles

Various classes of metal-based nanoparticles such as gold (Figure 1g), silver, carbon nanotubes, and quantum dots have been investigated as drug delivery tools in NSCLC therapy. The exponential growth in metal-based nanoparticles’ research is primarily due to their acceptable biocompatibility and ease of size manipulation and surface modification. Their visible light extinction properties have made them suitable for intracellular tracking [65].

Graphene (Figure 1h), a carbon monolayer arranged in a hexagonal honeycomb lattice, is also gaining significant attention owing to its superior drug loading capacity consisting of pi–pi stacking between the graphene sheet [67]. Nonetheless, there is still a lack of comprehensive understanding of the physicochemical property of graphene to maximize its usage in drug delivery systems [68]. Hoseini-Ghahfarokhi et al. provided a detailed review on the application of graphene as a drug delivery platform [69].

## 3. Routes of Administration of NDDS for NSCLC

Delivering NDDS for lung-related diseases has been challenging, but it holds significant potential. Numerous efforts have been reported in the literature to determine the best approach, route, material, and technique to achieve the desired therapeutic outcomes. In general, lung targeted delivery may be achieved through localized (inhalation) or systemic (intravenous) administration. Systemic administration has been the method of choice to ease delivery, but it causes several drawbacks such as sub-optimal concentration of drug at the site of lung cancer and toxicities on healthy cells [70].

Localized delivery is nevertheless a preferable approach as it lowers the occurrences of adverse reactions that may happen due to systemic distribution. The inhalation route of administration is not only useful for its local effect, but may also contribute to the systemic efficacy of drugs as they may also accumulate in the lymphatic circulation following administration [71]. As NDDS can reduce the biotoxicities of drugs through encapsulation, in vivo studies have shown the ability of pulmonary-administered NDDS to reduce the systemic toxicities of drugs, such as doxorubicin [72,73] and cisplatin [74]. The EPR effect may not be applicable in the pulmonary delivery of NDDS. However, the passive targeting method does play a role, besides the function of endocytosis that increases the likelihood of drug accumulation in cancer cells. Researchers have also investigated an active targeting method in lung delivery of NDDS. Tseng et al. reported that bEGF-decorated NDDS was internalized by EGFR-expressed tumor efficiently and a high dose of cisplatin was successfully achieved in the cancerous lung cells in animal models [75].

There are specific challenges that need to be considered to ensure an efficient pulmonary delivery of NDDS. The structure of the respiratory system and lung clearance mechanism may complicate the effort to ensure a sufficient amount of drug-encapsulated NDDS are accumulated at the cancer site [76,77,78]. The size of NDDS is of concern, as particles in the nanometer range are usually expelled during normal breathing. This reduces the possibility of NDDS retention in the lung. In addition, the design of nanoparticles should be made precisely to allow the deposition and release of the encapsulated drugs on cancer cells, whilst minimizing the exposure towards healthy cells. This will reduce the risk of toxicities whilst improving the treatment efficacy. Several approaches have been explored, as reported in the literature, such as pH-triggered release of drugs from NDDS [79], that will ensure the release of encapsulated drugs in a low-pH environment, which is commonly associated with the microenvironment of the surrounding cancer cells. Understanding the microenvironment is hence very important as a guide to designing a suitable NDDS for cancer targeting, in this case for lung cancer through pulmonary administration.

## 4. Cellular Evaluation of Drug Delivery System for Lung Cancer

Emerging evidence has highlighted the usefulness of nanoparticles as drug carriers, especially in cancer targeting, due to their ability to deliver various drugs with diverse characteristics in nature. However, it remains a challenge to choose the most suitable approach to closely characterize and evaluate the efficacy and safety profiles of nanomedicines in conditions that closely reflect the physiological complexity. It should be noted that the findings from preclinical studies may not represent the actual clinical outcomes of the developed nanomedicines. Herein, the cellular evaluation of nanomedicines against NSCLC lung cancer cells is reviewed based on the available data in the literature. Different types of cell lines have been reported as models to aid in the understanding of nanoformulation activity and effectiveness at the cellular level. This section will describe in detail the type of cell lines and the suitability of the cells to give the information needed in the evaluation of nanoformulations.

### 4.1. Lung Cancer Cell Lines

Since the 1970s, over 200 lung cancer cell lines encompassing many of the different histological subtypes of the disease have been established. Despite the fact that they have been established many years ago, they often retain characteristics of the original tumor they were derived from [80]. The number of individual lung cancer cell lines is almost the largest amongst epithelial cancer cell types, and 20% of cancer cell lines in the Sanger database are of lung cancer origin [81]. The selection of a suitable cell line to evaluate a specific aim of the developed nanocarrier is essential and overlooking this point might lead to a variety of misinterpreted data. This situation may arise due to some complications such as possible selection of minor tumor subpopulations that do not have the characteristics of the original population; possible acceleration of genomic instability; the absence of stromal, immune, and inflammatory cells; and the vascularization of, as well as the difficulty of evaluating, metastatic potential [82]. On the other hand, the proper selection of a cell line with the appropriate preparation can give the benefits of the cells’ limitless replicative ability, availability of in vivo and in vitro tests for the evaluation of invasiveness and tumorigenicity, possible identification of specific genetic, epigenetic and cytogenetic changes, the ability to determine specific environmental conditions for optimal growth, and the development of models to study multistage pathogenesis [83].

#### 4.1.1. Epithelial Cell Culture

Epithelial lung cells are valuable tools for the study of multi-stage lung cancer pathogenesis. For research, two types of culture models are available, namely primary cultured cells and immortalized cell lines.

##### Primary Cell-Based Models

Generally, primary cell cultures retain the morphological and biochemical characteristics of the tumor from which they were originally derived [84]. The primary cell culture highly represents the native epithelia, as they are isolated directly from the tissue, thus having more similarity in the cell characteristics. However, in the case of lung cancer cells, this cell culture is more difficult to obtain due to the lack of availability of normal human airway tissue; additionally, it is time-consuming to maintain [85]. Its reduced lifespan, higher cost, and low reproducibility make it less preferred as a permeation model compared to immortalized cell lines. Highly differentiated primary cell lines are commercially available in the form of human 3D in vitro respiratory tissue models such as EpiAirwayTM (MatTek Corporation, Ashland, MA, USA) and MucilAirTM (Epithelix SAS, Archamps, France). Both cell models are cultured to form multi-layered, well-differentiated models that closely resemble the respiratory tracts’ epithelial tissues. Results have shown that primary lung cancer cell culture is possible to be obtained from percutaneous puncture, providing a significant biological source to assess and investigate lung cancer’s molecular mechanisms [84]. Furthermore, primary cultures preserve cancer cells with stem-like phenotypes, an advantage not always offered by cell lines. Thus, this ex vivo system represents an important cancer research tool, but samples require correct manipulation to maximize their translational value. The use of these cells, however, requires ethics approval and is dependent on the availability of surgical material [86].

Primary cell immortalization can be performed using in vivo models, usually of mice. The microenvironment of living tissue promotes cancer cells’ growth, mainly due to the high number of epithelial components and the subsequent crosstalk of the tumor [87]. The combination of 3D technologies and primary cultures represents one of the in vitro models that comes closest to reproducing the tumor’s actual pathophysiological features [88] in terms of gene expression profiles, cellular signaling pathways, and the cell–cell and cell–extracellular matrix interactions [89].

##### Immortalized Cells-Based Models

In vitro cultures of immortalized cell lines isolated from tumors have been used as model systems in cancer for at least 65 years [90]. Understanding drug transport mechanisms in the human lung is a crucial issue in pulmonary drug discovery and development. For this purpose, there is an increasing interest in immortalized lung cell lines as alternatives to primary cultured lung cells, along with the need to provide a comprehensive quantification of protein expressions in immortalized lung cell lines [91]. Many cancer cells isolated from tumors are immortal in culture and are simple to maintain and not limited to passages [92]. Although these types of cells are often associated with a loss of ability to differentiate and showing less similarity in the biochemical characteristics of the original tissue, they have often been used as a model in the permeation study, as they are more reproducible and homogenous, easier to obtain and maintain, and relatively cheaper compared to primary cells [93]. Several types of cell lines that were transformed or derived from lung tumors are being used to develop models of epithelial barriers of the respiratory tract (Table 1).

Immortalized cell-based models can be composed of solely immortalized cells of two primary pulmonary cell types, namely bronchial and alveolar cell lines. The commonly used bronchial cell lines include Calu-3. This cell line can be cultured under different conditions, namely air–liquid and liquid–liquid interfaces, resulting in the formation of tight epithelia in both cell cultures [95]. However, appropriate culture conditions are imperative in the development of the epithelial cell model as the air–liquid interface was shown to promote proper cell differentiation and caused an increase in the expression of drug transporters, while the liquid–liquid interface caused an increase in the transepithelial electrical resistance (TEER). Furthermore, conditionally immortal cell lines derived from C57/BL6 mice might serve as an excellent illustrative example of an appropriate model system for cancer prevention studies [92].

The Calu-3 cell line can be used to assess formulation transportation in the lung epithelial cell such as in the evaluation of intracellular uptake and transport capability of a liposomal powder formulation loaded with ciprofloxacin and colistin. A study showed that the co-loaded liposomes resulted in a lower transport capability of both drugs across the Calu-3 cell monolayer, resulting in an accumulation in the cell. Since the treatment was aimed at respiratory tract infections, drug retention in the cells is expected to be beneficial [96].

In utilizing alveolar cell lines, the human lung alveolar adenocarcinoma cell line (A549) was often used as a model as it mimics the morphological and biochemical properties of human lung cells and secretes surfactant protein [97]. Monolayers of these cells were developed on cell culture inserts and used as lung barrier models to predict the inhalational toxicity of nanoparticles towards the lung. However, this cell line is unsuitable for drug permeability experiments due to its lack of functional tight junctions. The intrinsic properties limit its function as an effective in vitro model; thus, co-cultures of these cell lines with other types of cell lines are often made to strengthen the model. Though monoculture models are very convenient due to their simplicity, lack of interaction, and co-operative function between different cell types, they may not portray the lung’s real condition. Most of the time, the complementary cells are from the immune or vascular systems or support cells. In one study, co-culture of the A549 cell with macrophages was performed to evaluate the role of immune cells in ZnO nanoparticles’ internalization [98].

Co-culture of the A549 cell line with two different types of cells to create a more complex system resembling the human airways was reported in a study by Wang and co-workers [97]. The A549 cells were cultured with human differentiated monocytes (THP-1) and human umbilical vein endothelial cells (EA. hy926) in an air–liquid interface in vitro exposure system. The co-culture model was arranged so that the architecture mimics the anatomy of the alveolar region, with the endothelial cells on the basal side of the transwell and epithelial cells and macrophages on the apical side of the transwell. This model has shown to form an enhanced tight junction, improving the atypical tight junction associated with the monoculture model of the A549 cells. Enhanced biological responses towards airborne engineered nanomaterials (ENMs) were also achieved, reflecting an ROS production pattern closely related to human bronchial epithelial cells (HBECs) [97].

The monolayers of the single- or co-cultured cell lines can be grown under different conditions, namely liquid–liquid interface (LLI) and air–liquid interface (ALI) conditions. The former is a conventional method to evaluate nanoparticles’ dissolution rate but may not be suitable for assessing nanoparticles intended for inhalation purposes due to the extremely thin aqueous layers in the lungs [94]. Different culture conditions may also influence the properties of the cultured monolayers. For instance, a monolayer formed using Calu-3 cells was able to produce a mucus layer when cultivated under the ALI condition, providing a more similar model to the in vivo condition of the bronchial epithelium, compared to cells cultured in LLI condition [99].

### 4.2. Assays Used in Lung Cancer Drug Delivery

#### 4.2.1. Cytotoxicity Assays

Cell-based in vitro assays are routinely used to determine direct cytotoxic or antiproliferative effects of test substances on various cell lines. These assays have been used in the characterization of nanoformulations for NSCLC treatment including liposomes [100,101], solid lipid nanoparticles [102,103], polymeric nanoparticles [104,105], and metal-based nanoparticles [106]. The current antiproliferative assay principles are based on cellular enzyme activity, cellular ATP levels, protein or DNA interaction, and membrane integrity, which are known characteristics of viable and non-viable cells [107]. The use of these assays has been rapidly increasing over the years due to their rapid, economical, and large sample testing capacity, besides eliminating the need for animal studies. In the case of nanomaterial safety assessments, these in vitro assays are crucial for discriminating between safe and hazardous nanoparticles and investigating the specific mechanism pathways of internalization and uptake mechanisms and causes of cell death. On the other hand, investigations of the enhanced cytotoxic properties of nanoparticle drug delivery systems loaded with known anticancer agents would entail a careful selection of assay and cautious interpretation of the obtained results. As such, mechanism-based cytotoxicity assays may also be carried out in conjunction with the standard assays.

A number of antiproliferative assays with different principles have been developed for the cytotoxicity screening of nanomaterials, as shown in Table 2. However, there is a need for careful consideration of the type of nanomaterials, size, and surface modifications to minimize any possible interaction with the assay components. The classification of these assays is usually based on the endpoint measurements used in the detection methods. The tetrazolium salt-based assay is one of the most commonly used assays for cell viability and proliferation screening [108]. The tetrazolium salt includes MTT (3-(4,5-dimethylthiazol-2-yl)-2,5-diphenyltetrazolium bromide), MTS (3-(4,5-dimethylthiazol-2-yl)-5-(3-carboxymethoxyphenyl)-2-(4-sulfophenyl)-2H-tetrazolium), XTT (sodium 2,3-bis-(2-methoxy-4-nitro-5-sulfophenyl)-2H-tetrazolium-5-carboxanilide), and WST-1 (4-(3-(4-iodophenyl)-2-(4-nitrophenyl)-2H-5-tetrazolio)-1,3-benzene disulfonate). The reduction of tetrazolium salt to formazan product is facilitated by dehydrogenases localized in the mitochondria of viable cells [109]. The main difference between MTT and the other assays is that MTT reduction occurred intracellularly, and solubilization is required to measure formazan absorbance. On the other hand, MTS, XTT, and WST-1 are water-soluble, negatively charged, and do not readily penetrate cells. These substrates are typically used with an intermediate electron acceptor that can transfer electrons from the cytoplasm or plasma membrane to facilitate the reduction of the tetrazolium into soluble formazan, thus eliminating the need for the solubilization step [110].

MTT assay was first described by Mosmann [111] and subsequently improved by several other investigators [129,130,131]. MTT is a sensitive and reliable substrate indicator of cellular metabolic activity and is preferred over the other methods measuring this endpoint, such as the ATP and 3H-thymidine incorporation assay [132]. The substrate is briefly added to cells in culture, usually at a final concentration of 0.2–0.5 mg/mL and incubated for 1 to 4 h. The MTT assay measures the growth rate of cells by virtue of a linear relationship between cell activity and absorbance [108].

It is worthwhile to note that MTT reduction is readily affected by metabolic and other factors such as the composition of cell medium, which may, in turn, substantially affect the quantitation of cell viability [131]. MTT formazan formation varies significantly among cell lines in both the kinetics of its formation and the degree of saturability exhibited [131]. Additionally, metabolism and exocytosis of MTT are found to activate apoptosis-related factors such as caspase-3 and caspase-8 or accelerate the leakage of cell contents after the appearance of MTT formazan crystals, causing cell damage [133]. The addition of exogenous compounds with reduction properties can interfere with the tetrazolium-based assays and produce false-positive results. Chemicals such as ascorbic acid, dithiothreitol, mercaptoethanol, L-cysteine, and other bioactive compounds can also reduce tetrazolium salts non-enzymatically and lead to increased absorbance values detected in the assay wells [134,135,136,137,138]. Hence, in the evaluation of nanoformulation cytotoxicity in NSCLC cell lines, nanoparticles containing active ingredients with reducing and antioxidant abilities may be likely to interfere with the results.

XTT, including MTS and WST-1, also presents several problems associated with high-flux drug screening, including the inability of many cell lines to metabolize the tetrazolium in the absence of an added electron transfer reagent such a phenazine methosulfate [139]. However, MTS is more soluble and nontoxic than XTT, which allows cells to be used for further evaluation [140]. The downside with MTS solubility is that it may be susceptible to colorimetric interference since the reaction is carried out in a one-step manner in the presence of traces of colored test compounds. This limitation may also apply to nanoparticles loaded with colored compounds. Since the net negative charge of these soluble tetrazolium salts prevents their intracellular uptake and facilitates extracellular reduction, nanoparticles interfering with the cell membrane may also affect their reduction. The electron transport process may be compromised, giving false-negative results. Hence, in such cases, a positively charged tetrazolium salt such as MTT may be desirable and provide a more reliable evaluation of cytotoxicity [132].

The WST-1 substrate offers several advantages over MTT and XTT, including water-solubility and greater stability and sensitivity [141]. However, a recent study has highlighted misleading cell viability results with WST-1 [114]. Apparently, endothelial cells exposed to Mn particles (Mn alone or Fe–Mn alloy from 50 to 1600 μg/mL) were severely damaged based on WST-1 assay, but not the ATP content assay. Further investigations revealed that Mn particles interfere with the reduction of the WST-1 to formazan, possibly via direct binding, giving false cytotoxicity results [114]. Hence, WST-1 assay or perhaps other tetrazolium salt-based assays may not be suitable to evaluate the in vitro cytotoxicity of Mn-containing materials.

Several studies have highlighted misleading cell viability results when using tetrazolium salts to evaluate cell viability following treatment with nanomaterials. Wörle-Knirsch and co-workers have demonstrated that single-walled carbon nanotubes bind to MTT-formazan crystals, producing false cytotoxicity results [142]. Data from A549 cells incubated with carbon nanotubes produced a significant cytotoxic effect when using MTT assay after 24 h, whereas the same treatment detected with WST-1, LDH, FACS-assisted mitochondrial membrane potential determination, and Annexin-V/PI staining revealed no cytotoxicity. The carbon nanotubes appear to interact with some tetrazolium salts such as MTT but not with others (such as WST-1, INT, XTT) [142]. Nanomaterials made from carbon black alone could interact with MTT dye and cause false cytotoxicity results [143]. Corroded Mg is found to convert tetrazolium salts to formazan, leading to a higher background and falsifying the results of cell viability, indicating that tetrazolium-based assays are not a useful tool to evaluate the cytotoxicity of Mg-based nanoparticles in static in vitro assays [143]. In another study, MTT is a potential confounder in nanoparticle toxicity testing for trisilanol phenyl and trisilanol isooctyl polyhedral oligomeric silsesquioxane particles [144]. In addition, cadmium selenium (CdSe 100) nanoparticles and helical rosette nanotubes (RNT 100) are found to interfere with the MTS assay [118]. When negatively charged CdSe were added to the MTS assay, a significant over-estimation of cells was observed [118]. An important consideration of these toxicity test systems is that they rely on absorbance, fluorescence, or luminescence changes of the final product. Hence, certain nanoparticles made from metals such as gold (Au) may absorb light in the visible region (~520 nm) and interfere with the endpoint measurements [145].

The measurement of ATP in cytotoxicity testing is based on the luciferin–luciferase bioluminescent reaction, which requires luciferase enzyme, luciferin and ATP, magnesium (or other divalent cations), and oxygen. ATP concentration declines rapidly when cells undergo cell death; hence, ATP levels are a reliable indicator of cytotoxicity effects [122]. ATP in the viable cells will react with luciferin in the presence of luciferase and form luciferyl adenylate, which is then oxidized to oxyluciferin and produces luminescence. The emitted light is quantified with a luminescent reader, whereby the measured luminescence signal is directly proportional to the amount of ATP and representative of the live cells present in the sample [146]. Generally, the ATP assay is rapid, sensitive, and less prone to artifacts compared to other viability assays. An ATP-based luminescent viability assay combined with microscopic imaging is also found to be a more reliable screening tool as compared with measuring the therapeutic effect in glioma cell lines and glioma stem-like cells [147]. However, certain nanoparticles may affect the assay, for example, silica nanoparticles are found to interfere with ATP bioluminescence, producing low ATP measurement, which can be mistakenly assessed as a result of significant inhibition [146].

The LDH assay principle is typically based on cell membrane integrity and monitoring the release of lactate dehydrogenase from compromised cells [125]. LDH assay endpoints can be either colorimetric or fluorometric. Experimentally, LDH activity typically involves a coupled enzymatic reaction, where LDH oxidizes lactate to pyruvate, which subsequently reacts with iodonitrotetrazolium chloride (INT) to form formazan, which can be measured at 490 nm [148,149]. The amount of formazan is directly correlated with the amount of LDH release and cell death. Generally, the LDH release assay is reliable, rapid, and straightforward [149]. However, the application of drugs or compounds intended to trigger LDH release requires careful optimization to achieve a sufficient window to quantify cytoprotection without confounding the assay by differences in cell proliferation [148]. Furthermore, LDH may be taken back into the cells or metabolized after prolonged insults. Additionally, copper (Cu-40) and silver (Ag-35) nanoparticles are found to interfere with the LDH assay by inactivating LDH [150,151]. Similarly, titanium dioxide (TiO_2_-25) nanoparticles are also capable of adsorbing LDH molecules, affecting the LDH assay [152]. ZnO interacts with LDH, resulting in less accurate cytotoxic measurements [151]. Adsorption of LDH on the carbon nanotube surfaces has also contributed to the interferences in the LDH assay results [153].

The trypan blue exclusion assay principle is similar to the LDH assay, except that the trypan blue assay involved microscopy examination of cells. This simple assay requires the use of a hemocytometer and the calculation of cells stained with the blue dye indicating dead cells. [127]. Hence, trypan blue is negatively charged and will not be taken up by cells with an intact cell membrane. Although this assay may be useful for daily laboratory routines, it requires the harvesting of cells by trypsin, making it inconvenient for high throughput or multiple assay screenings [154]. Thus, the trypan blue exclusion method is usually not robust enough to determine cytotoxic properties of the test substances, including nanoparticles. A preliminary determination of possible enhancement of cytotoxicity activities of experimental nanoparticles loaded with anticancer agents is indispensable and very useful. However, as mentioned above, some of these assay components may not be desirable for certain nanomaterials because of dye–nanoparticle interactions. Additionally, most assays destroy the cells at the end of the experiment and are incompatible with the kinetic or real-time analyses of compound toxicity, which are both dose- and time-dependent [128].

A recent innovative strategy to measure cell viability in real time was developed to overcome such limitations. The bioluminescent assay is based on the cellular capacity to reduce a luciferase prosubstrate to a form that is then rapidly utilized by the luciferase enzyme present in the assay [128]. Since the reduced probe is rapidly utilized by luciferase, it does not accumulate. Hence, a steady-state signal is maintained, which correlates to the number of viable cells present at a given time. The clear advantage of this assay is that the reagents are nontoxic to cells and the turnover of the prosubstrate is slow; thus, continuous reads can be obtained over an extended period (72 h) [128]. This nonlytic homogeneous bioluminescent assay also provides rapid, high-throughput, and extensive multiplexing capability. However, since the assay involves luminescence, there is still a risk of possible interaction with nanomaterials, and more studies are certainly needed to prove this point.

In nanoparticle-based drug delivery system, parameters such as the nanoparticle size, concentration, coating, use of GRAS excipients, agglomeration, surface corona, charge, and hydrophobicity/hydrophilicity significantly affect the biological responses at the cellular level [155]. Thus, optimizing these parameters and ensuring the robustness of the assay are essential for compatible physiological results. Furthermore, other factors such as drug concentration, time of exposure to the drug, length of the assay, and cell density could also be considered limitations if not properly optimized before conducting the assay [156].

Cytotoxicity assays are crucial preliminary assays to determine the safety profile of nanoparticles using cell lines. In fact, the EU NanoSafety Cluster group has suggested that at least four methods of determining cytotoxicity should be used in order to obtain a reliable safety profile for novel nanomaterials [157]. As in the case of nanoparticles loaded with anticancer agents, these basic assays are important to gauge possible enhancement or improvement of cytotoxicity or antitumor activities of studied anticancer compounds formulated as nanoparticles. However, it is important to note that the ultimate endpoints of these assays are dead cells and hence are usually not comprehensive enough to characterize the mode of cell death. Some of the more useful assays that may complement the basic antiproliferation assays in anticancer drug screening include the TdT dUTP nick end labeling (TUNEL) assay, which specifically detects fragmented DNA, a feature of both programmed necrosis and apoptosis; the Comet assay, which uses single-cell gel electrophoresis to detect DNA strand breaks; the apostain-based assay, which detects chromatin changes; the ROS production; the annexin V-FITC staining using a flow cytometry; and other assays that monitor changes in the gene or protein expression in cells, including reverse transcriptase-quantitative polymerase chain reaction (RT-qPCR)-, microarray-, and PCR-array-based assays. These assays are often used in the evaluation of anticancer drug-loaded nanoparticles against lung cancer cell lines such as A549 and H1299 [158,159,160,161].

#### 4.2.2. Permeability and In Vitro Cellular Uptake Assay

**Figure 2:**
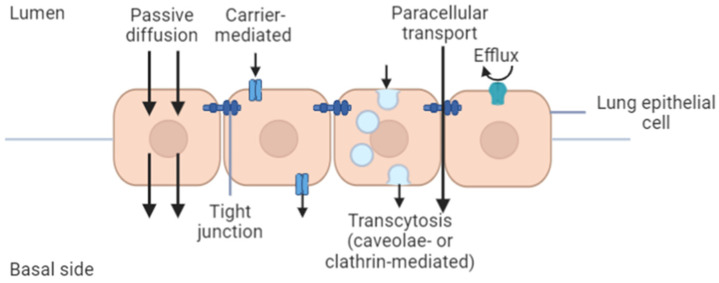
Schematic diagram of different absorption mechanisms in lung epithelium (created using BioRender.com, accessed on 2 April 2021).

Indeed, the barriers involved in the administration route must be fully understood to ensure optimal delivery of the drugs to the cancerous site. In vitro techniques are imperative to assess the fate of the formulated drug in the lung including binding, transport, uptake, metabolism, and any toxicity related to pulmonary route administration. Particularly, in vitro models for predicting the permeability of drugs or nanoparticles would offer several advantages compared to the in vivo studies, as the former offer simplicity and reproducibility, are less expensive, and do not require ethical considerations [162]. The in vitro model also allows the manipulation of specific cellular pathways to provide insight for a better understanding of in vivo systems. The safety and efficacy of therapeutic compounds or formulations can be assessed based on the permeability and absorption mechanism derived from in vitro experiments [94]. In lung tissues, permeability through the pulmonary epithelium will determine the extent of nanoparticle distribution in the body, whether they remain in the lung or are redistributed through the systemic circulation. If the formulation is intended for local delivery, low permeation through the epithelium is desirable to increase the nanoparticles’ concentration in lung tissues, allowing the drug to exert its therapeutic effect locally. In contrast, high permeation through the lung epithelium is advantageous if the formulation is intended for systemic delivery [93]. The permeation profile depends on a drug or nanoparticle’s physicochemical properties, such that hydrophilic molecules cross the epithelial membrane via paracellular and carrier-mediated pathways while lipophilic molecules cross the membrane via the transcellular pathway (Figure 2) [93,162].

Vesicular transcytosis happens in the presence of caveolae (in pulmonary endothelial cells and alveolar type I epithelial cells) and clathrin-coated pits (in alveolar type I and type II cells) [163]. The vesicular transportation is size-dependent, with particles <200 nm being internalized by the clathrin-coated pits while internalization of bigger particles (200–1000 nm) relies on the caveolae-mediated endocytosis [164]. The presence of efflux transporters in the pulmonary epithelium may alter the residence time and absorption rate of certain drugs, which could be favorable for locally acting delivery systems. There are three major in vitro models established to resemble the epithelial biological barriers of the human lung. The models can be ranked according to their decreasing complexity as follows: isolated perfused organ > isolated tissue > epithelial cell culture. Owing to its complexity, the isolated perfused organ model has the most closeness to the in vivo conditions of the human lung [85].

In vitro and in vivo studies suggest that active targeting nanoparticles will increase selectivity in the cellular uptake and/or cytotoxicity over the conventional chemotherapeutic drugs and non-targeted nanoparticle platform, particularly enhancing drug efficacy and safety [165]. Ligand selection on the nanoparticles’ surface plays a critical role in their cellular uptake [166]. Moreover, particles’ cellular uptake is a particle-size-dependent phenomenon and has been shown to increase with decreasing particle size [71]. Thus, it is imperative to visualize or quantify the number of nanoparticles taken up by the targeted or untargeted cells to determine their efficiency and safety, respectively. The method of choice, mainly spectroscopic and imaging methods, highly depends on the characteristics of the particles including the surface characteristics, size, shape, and spectroscopic properties (e.g., fluorescence and scattering) [167]. Several imaging techniques have been applied to visualize the uptake of nanoparticles in the NSCLC cell lines including the confocal fluorescence microscopy [168,169,170], transmission electron microscopy [168], and flow cytometry [171]. The former is a commonly used technique based on qualitative determination using fluorescence-based nanoparticles. The fluorescence signals from the nanoparticles could be derived from an intrinsic property of the loaded drug (e.g., doxorubicin) [169] or fluorescence tags (e.g., fluorescein isothiocyanate) [168]. Nonetheless, the fluorescence tags may affect the uptake processes and may cause bleaching of fluorophores, quenching, and induction of phototoxic reaction [172].

For a better understanding of the uptake process, quantification of the nanoparticles in the cells would be valuable. However, the small particle size and low mass of the delivery system limit the sensitivity and resolution of certain techniques. Inductively coupled plasma (ICP)-based spectroscopic techniques have been used in the quantification of the nanoparticle uptake in the NSCLC cell lines including ICP-mass spectrometry (ICP-MS) [173,174] as well as ICP-optical emission spectroscopy (ICP-OES) [175]. Although these techniques have a very sensitive detection range (ppt to ppm), they could only determine the nanoparticle mass in a cell population, not in a single cell. Nonetheless, the nanoparticle uptake of a single cell can be extrapolated by determining the cell number in one population during the cell culture assay [167].

#### 4.2.3. Permeability Assays

##### Isolated Tissue Model

Animal models have been used in the evaluation of toxicological, allergic, pro-inflammatory, and immunological properties of pharmaceutical products or formulations in pre-clinical studies. However, utilization of ex vivo human systems has gained increasing attention for high-throughput screening of potential toxicological and allergic reactions of the lung towards pharmaceutical formulations, as they can overcome some limitations related to the animal models, including high cost and time spent. One of the most common tissue models is precision-cut lung slices (PCLS), which can be cultured from the explanted healthy or diseased lung of human or animal models, with a more mechanistic resemblance to multiple regions of the lung [176]. This model is more comprehensive for assessing local responses such that they maintain the structural integrity of the tissue and its cell populations while reflecting the extracellular matrix associated with the disease. The model is created by cutting the lung tissue using microtomes or tissue slicers with enhanced precision and reproducible thickness, hence the technique being named precision-cut tissue slices [177].

Placke and Fisher in 1987 had successfully developed a method to obtain PCLS, which previously imposed a technical challenge for soft tissues such as lungs [178]. The method involves the infusion of heated liquid agarose into the airways of hamster and rat lungs. The solution, which was solidified at 4 °C, helps to maintain the inflated states of the lung, thus preventing the collapse of airways and alveoli during the slicing process. The PCLS are then maintained ex vivo in multi-well plates containing the culture medium, which are optimized to allow the viability of the tissue without compromising its cytotoxic, inflammatory, and immune responses against selected stimuli. Generally, PCLS can survive for a period of up to 7 days [179]. However, the cultivation period can be prolonged for up to ~14 days [180,181,182] or even 21 days [183] when cultured in appropriate media systems. Longer cultivation of PCLS is essential for chronic exposure experiments.

PCLS has been used to assess the toxicity and efficacy of biological agents or materials to the respiratory tract [184,185], including the siRNA-mediated RNA interference [186] and inhalable influenza vaccine [187]. It has a greater advantage over the cell line assay as it represents the actual condition of the lung, especially in disease conditions. In a study, PCLS prepared from bleomycin-treated mice was used as an ex vivo idiopathic pulmonary fibrosis model [188]. The model was used to evaluate compounds (i.e., ALK inhibitor SB525334 and nintedanib) that are used in the treatment of the disease, and it was shown to have an increased expression of fibrosis-related genes that are similar to the in vivo bleomycin model. In the application of lung cancer, a tissue slice model was prepared from isolated human tumor tissue slices and primary lung cancer cells from NSCLC patients [189]. The model was used to evaluate chitosan-coated poly(lactide-co-glycolide) nanoparticles containing an oligonucleotide, allowing the assessment of the nanoparticle penetration into the tumor tissues and its efficacy in inhibiting telomerase activity in the original condition of the solid tumor. Further, potential interactions between different cell types in the lung tissue may be better simulated in the PCLS model. In a toxicity evaluation of solid lipid nanoparticles’ formulation [190], a difference in the level of toxicity was exhibited in the PCLS when compared to the A549 cell line. Lower cytotoxicity in the PCLS was possibly due to the potential interactions between the different cell types present and their susceptibility to chemical stimuli in the PCLS model.

##### Cell Line-Based Permeability

Being cultured in ALI conditions, the multi-layered monocultures were demonstrated to be compatible with testing drugs administered as a liquid aerosol by a clinical nebulizer, offering an advantage over 3D tumor spheroids [191]. There are distinct differences in morphology and permeability when Calu-3 cells are grown using an air interface culture (AIC) or liquid covered culture (LCC). Studies have shown that cells cultured using the AIC generate a more morphologically representative structure of the in vivo airway epithelium than cells cultured on LCC in terms of ultrastructure, secretory components, and electrical resistance. Additionally, AIC produces a thick mucus layer on the apical surface, whereas in the LCC model, only secretory vesicles could be observed. Finally, measurement of TEER, routinely used to determine the integrity and permeability of the epithelial cells, demonstrated less restrictive tight junctions in the AIC model compared to the LCC model [192].

#### 4.2.4. Three-Dimensional Cell Models for Nanomedicine Research 

To date, there is a variety of 3D cell models available that can be employed to design and evaluate the efficiency of drug nanocarriers. These models enable the researchers to study the specific aspects of the nanomedicine behavior in vivo, although none of these models can recapitulate the exact complex tumor micro-environment. Among the common 3D multicellular in vitro tissue systems are organoid and spheroid systems [77]. They can be derived from the pluripotent (embryonic or induced) or adult stem cells from various organs, enabling further investigation of various aspects of a particular organ in the tissue culture dish without the presence of a complex in vivo environment [193,194]. This 3D culture system has been reported to significantly enhance the cell–extracellular matrix interaction besides improving the survival, proliferation, differentiation, and responses of the target cells [195,196]. The toxicity of neutral G5-OH nanoparticles tested in organoid is comparable to and closely reflects the toxicity markers reported in rodent nephrotoxicity models exposed to the similar nanoparticles [197]. Similarly, selective targeting of CD44-overexpressing NSCLC cells by hyaluronan-based nanoparticles has been observed in 2D and 3D cultures and in in vivo orthotopic lung cancer models. The nanoparticles provided active targeting partially mediated by CD44 and were also found to demonstrate less toxicity with improved antitumor efficiency [76,77,78]. Despite its robustness and functional relevance to native tissues and organs, the dynamic transport of nanotherapeutics in a 3D culture system is often overlooked. There is a possibility of alteration in kinetics and nanoparticle–cell interactions, which are dependent on the sedimentation and diffusion velocities of the nanoparticles used. Hence, these factors must be meticulously considered when evaluating cellular uptake with large and/or heavy nanoparticles [198].

Another advanced 3D cell model is the microfluidics-based organ-on-a-chip (Organ Chip), which has recently received increased attention as a promising platform as this cell model accurately replicates the microenvironments of native tissues and various tissue-tissue interactions [199]. It simulates the tissue-level or organ-level physiology, which is not possible with conventional 2D or 3D static culture systems via continuous perfusion of the chambers containing living cells using a microfluidic cell culture device. A novel 3D human lung-on-a-chip model that recreates the organ-level structure and functions of the human lung has been shown to effectively evaluate the pulmonary toxicity of nanoparticles [197]. It has been acknowledged that the Lung Chip models offer greater potential beyond just toxicity testing, as they can be utilized to evaluate various nanodiagnostics and nanotherapeutics relevant for lung cancer. Nevertheless, the development of an organ chip system is laborious and challenging, since there is a possibility of the interaction between nanomedicine and microfluidics systems as well as the difficulty to initiate and maintain the primary cells used in these systems. Additionally, achieving real-time and continuous monitoring of the biological effects of nanomedicine and the requirement to standardize the Organ Chip to accurately evaluate the pharmacokinetic profiles of the nanomedicine remain a challenge [199].

## 5. Perspective: Challenges and Opportunity

Notwithstanding the advantages of cell lines in evaluating the efficiency of delivering nanomedicine for lung cancer treatment, questions have been raised about their reliability since not all lung cancer subtypes are well represented by these cell-based experimental models [200]. The stromal, vascular, and inflammatory cells, which are fundamental components in the development of local tumors as well as in the metastasis and angiogenesis during the various lung cancer stages, are also lacking in tumor-derived cell lines as opposed to the living tumor tissues [82]. The inaccuracy of cell morphology and functionality, as well as the lack of a physiological matrix-like microenvironment exemplified in 2D cell models, remains a major challenge in utilizing in vitro models to evaluate the efficacy and safety profile of nanomedicine. These factors indirectly limit their ability to recapitulate the appropriate levels of in vivo cellular responses [201]. This issue, however, can be minimized by employing 3D models that have been shown to fundamentally bridge between in vitro and in vivo studies, expanding nanoparticle translation to in vivo and clinical stages in NSCLC research. Despite the advancement of 3D culture systems, 2D culture models remain the most employed approach and standard practice in nanomedicine research as they are less laborious and easier to set up [202].

The outcome of a seemingly simple cytotoxicity assay might be highly dependent upon the respective protocol and the test substance used. Various factors are known to contribute to aberrant results, such as the physical interactions between the nanomaterials used to manufacture the nanoparticles and the components in the assays (dye, surfactant etc.), changes in cellular activities involved in redox reactions in response to the nanomaterials, and many other unknown factors that may influence the determination of the cytotoxicity profile of nano-particulate systems [203]. Perhaps it is a good idea that any interference between raw nanomaterials and assay components should be thoroughly evaluated prior to the commencement of a cytotoxicity assay to establish compatibility and to obtain some baseline data. On the other hand, well-defined and characterized nanoparticles should be used in these assays. This will ensure that the data obtained are reliable and not due to unforeseen errors.

In addition, higher concentrations of nanoparticles (>10 mg/L) appear to have a greater probability of interfering with assay function [118]. Hence, it would be plausible to limit or optimize the concentration of nanoparticles or the concentration of drug(s) entrapped in the nanoparticles [143]. Another consideration is that the final nanoparticle concentration present in the assay may be much lower than the experimental concentration due to biophysicochemical interactions that occur at the nano–bio interface, resulting in incomplete membrane translocation or binding to various biological components [204]. Generally, it is not possible to accurately predict how each nanoparticle and its contents will interact, react, or behave in any of the chosen assays. Reliance on a single assay could be risky and may provide either false positive or negative results. Hence, the establishment of baseline quality control, best practices, and comparative analyses of multiple assay platforms are indeed essential.

## 6. Conclusions

Pharmaceutical technology has been playing a vital role in medicine, as it allows the exploration of different materials and their combinations to prepare drug carriers, and to further endow these with better properties that enable them to reach the desired target site. Many questions arise around the topic of nanocarrier-based lung drug delivery systems. Scientists have given a plethora of responses in the attempt to address all the issues raised, finding alternatives and engineering adequate systems to fulfil requirements and needs. One of the concerns is always the fate of the drug particles once they reached the target site. In pulmonary drug delivery, the objective is sometimes to retain the drug in the lung, as happens in local delivery approaches, thus minimizing the systemic absorption. In other cases, an intravenous delivery route would aim for a systemic effect, and the carriers are engineered to avoid retention at the administration site, with a specific targeting agent attached that would eventually bring the nanocarrier to the lung for the desired therapeutic effect.

In vitro cell-culture-based models have been used for permeability screening and cell uptake study of drugs or formulations. Although some cell lines have limitations, continuous improvements have been made including co-culture with other cells to produce a model that can mimic the human lung epithelial barrier as closely as possible. These models are easy to exploit and do not involve ethical considerations related to animal use, as required in a general in vivo study. In a nutshell, the basis behind each decision made on an experimental model should be aligned to the research aims and relevance to human physiology and should be pertinently translated.

## Figures and Tables

**Table 1:** Examples of cell culture models used to simulate epithelial barriers of the respiratory tract [85,94].

Cell Types	Cell Line	Description
Bronchial	16HBE14o-	Human bronchial epithelial cell line (postcrisis large simian virus 40 large T-antigen transformed epithelial cell line).
	BEAS-2B	Human bronchial epithelial cell line (immortalized using adenovirus 12-simian virus 40 hybrid virus).
	Calu-3	Human sub-bronchial gland cell line (derived from a bronchial adenocarcinoma of a 25-year-old Caucasian man).
Alveolar	A549	Human alveolar lung adenocarcinoma cell line
	hAELVi	Primary alveolar epithelial cells (derived from human lung after surgery).

**Table 2:** Different cytotoxicity assays for the evaluation of nanoparticles.

Assays	Method of Detection	Description	Interaction with Nanoparticles
Tetrazolium based substrates: MTT, MTS, XTT, WST-1 assays	Colorimetric	NAD(P)H-dependent oxidoreductase or dehydrogenases in viable cells can reduce tetrazolium salt into purple-colored (MTT/MTS), orange-colored (XTT), or orange to purple (WST-1) formazan, which requires either solubilization/non-solubilization process prior to spectrophotometric analysis [111].	Carbon nanotubes (MTT) [112]
			Carbon black (MTT) [113]
			Mn (WST-1) [114]
			Mg (Tetrazolium salt)
			Polyhedral oligomeric
			Silsesquioxane (MTT) [115]
			Au (MTT) [116]
			CdSe (MTS) [117]
			helical rosette nanotubes (RNT) (MTS) [118]
Sulforhodamine B (SRB) assay	Colorimetric	SRB binds stoichiometrically to proteins under mild acidic conditions and can be extracted using basic conditions; thus, the amount of bound dye can be used as a proxy for cell mass [119].	Au or other metals [120]
	Fluorometric		
Alamar blue assay (resazurin)	Colorimetric	Metabolic activity of cells converts soluble resazurin dye into fluorescent resorufin with fluorescence emission [121].	CdSe [112]
	Fluorometric		TiO_2_ [112]
Adenosine triphosphate (ATP) assay	Colorimetric	ATP present in viable cells will react with luciferin in the presence of luciferase, producing luminescence as the end product [122].	Au [123] Silica [124]
	Fluorometric		
	Luminometric		
Lactate dehydrogenase (LDH) leakage assay	Colorimetric	Monitoring the release of lactate dehydrogenase from compromised cells [125].	Au, Cu, Ag, TiO_2_, ZnO [116,126]
	Fluorometric		Carbon nanotubes [112]
Trypan blue exclusion assay	Microscopy	Dye uptake in cells with compromised cell membrane [127].	
Real time assay(Glo™ reagents)	Bioluminometric	Real time monitoring of viable cells based on luciferase–substrate reaction [128].	
